# Periodontitis and Oral and Oropharyngeal Cancer Risk: A Systematic Review and Meta-Analysis with Exploratory Evidence on Tumor-Associated *Porphyromonas gingivalis*

**DOI:** 10.3390/dj14020080

**Published:** 2026-02-02

**Authors:** Luis Chauca-Bajaña, Bernarda Andrea Sánchez Arteaga, Andrea Ordóñez Balladares, María Isabel Romero Vasquez, Gustavo Javier Icaza Latorre, Carla Verenice Romo Olvera, Mauro Xavier Zambrano Matamoros, Byron Velásquez Ron

**Affiliations:** 1 College Dentistry, University of Guayaquil, Guayaquil 090101, Ecuador; luis.chaucab@ug.edu.ec (L.C.-B.);; 2 Universidad Bolivariana del Ecuador, Durán 092406, Ecuador; 3 Carrera de Odontología, Department Prosthesis Research, Universidad de Las Américas (UDLA), Quito 170102, Ecuador

**Keywords:** periodontitis, mouth neoplasms, oropharyngeal neoplasms, *Porphyromonas gingivalis*

## Abstract

**Background:** Periodontitis is a chronic inflammatory condition characterized by progressive destruction of tooth-supporting tissues and sustained microbial dysbiosis. Increasing evidence suggests that chronic oral inflammation may be associated with oral and oropharyngeal carcinogenesis, although findings across epidemiological and prognostic studies remain heterogeneous. **Objective:** To systematically evaluate the epidemiological association between clinically defined periodontitis and the risk of oral and/or oropharyngeal cancer, and to explore, in a distinct analytical component, the prognostic association between tumor-associated periodontal pathogens, particularly *Porphyromonas gingivalis*, and survival outcomes in affected patients. **Methods:** A systematic review and meta-analysis were conducted following PRISMA guidelines and registered in PROSPERO (CRD420251273975). Observational studies evaluating periodontitis and oral/oropharyngeal cancer risk (Arm 1) and prognostic studies assessing tumor-associated periodontal pathogens and survival outcomes (Arm 2) were identified through comprehensive database searches. Random-effects meta-analyses were performed to pool adjusted effect estimates. Risk of bias was assessed using the Newcastle–Ottawa Scale and the QUIPS tool. **Results:** Six observational studies were included in the epidemiological meta-analysis. Periodontitis was significantly associated with an increased risk of oral and/or oropharyngeal cancer (pooled HR = 2.14; 95% CI: 1.53–2.98), with substantial heterogeneity; trial sequential analysis supported the statistical robustness of this association. In the separate prognostic analysis, three studies evaluating intratumoral *Porphyromonas gingivalis* were included. A higher presence or expression of *P. gingivalis* was associated with poorer overall survival (HR = 2.89; 95% CI: 1.93–4.32), with no observed heterogeneity. Sensitivity and influence analyses confirmed the stability of these findings. **Conclusions:** This systematic review and meta-analysis demonstrate a consistent epidemiological association between periodontitis and an increased risk of oral and/or oropharyngeal cancer. In addition, exploratory prognostic evidence suggests that the presence of *Porphyromonas gingivalis* within tumor tissue may be associated with adverse survival outcomes. These findings should be interpreted as addressing distinct clinical and microbiological constructs, underscoring the need for further well-designed prospective and mechanistic studies.

## 1. Introduction

Periodontitis is a chronic, multifactorial inflammatory condition characterized by the progressive destruction of the tooth-supporting tissues, including the periodontal ligament, cementum, and alveolar bone [1,2]. It is estimated that more than 45% of the adult population presents some degree of periodontitis, and approximately 10–15% develops moderate-to-severe periodontitis, which is associated with persistent systemic inflammation [3,4]. Chronic inflammation has been widely recognized as a key mechanism in carcinogenesis, promoting tumor initiation, progression, and dissemination through complex immunological and molecular pathways [5,6].

Oral and oropharyngeal cancer represent a major global public health burden due to their high morbidity and mortality rates, particularly in low- and middle-income countries [7]. Although tobacco use, alcohol consumption, and human papillomavirus infection are well-established risk factors, increasing attention has been directed toward periodontitis as a potential factor associated with the development of these malignancies [8,9]. Early clinical studies reported a significant association between chronic periodontitis and tongue cancer, suggesting that periodontal tissue destruction may be linked to an increased risk of oral carcinogenesis [10].

Subsequently, several observational studies, including cohort and case–control designs, demonstrated that individuals with periodontitis have a higher risk of developing head and neck squamous cell carcinoma compared with periodontally healthy subjects, even after adjustment for major confounding factors such as smoking and alcohol consumption [11,12,13]. In addition, indicators of poor oral health, including tooth loss, inadequate oral hygiene, and a history of periodontitis, have been consistently associated with an increased risk of oral and oropharyngeal cancer across different populations [14,15].

From a biological perspective, periodontitis is characterized by microbial dysbiosis and sustained inflammatory responses, which may favor a pro-carcinogenic microenvironment within the oral cavity [16]. In this context, several studies have highlighted the potential role of specific periodontal pathogens, such as *Porphyromonas gingivalis*, in immune modulation, apoptosis evasion, and tumor progression in oral squamous cell carcinoma [17,18]. Moreover, the presence or increased burden of *Porphyromonas gingivalis* has been associated with poorer clinical outcomes and reduced overall survival in patients with oral and/or oropharyngeal cancer [19].

Despite the growing body of evidence, available studies show considerable heterogeneity in terms of study design, periodontitis definitions, and control of confounding variables, which complicates the overall interpretation of the association between periodontitis and oral and/or oropharyngeal cancer [20]. Therefore, the present systematic review and meta-analysis aimed to comprehensively evaluate the association between periodontitis and the risk of oral and/or oropharyngeal cancer, as well as to assess the prognostic impact of periodontal pathogens in affected patients.

## 2. Materials and Methods

**Figure 1:**
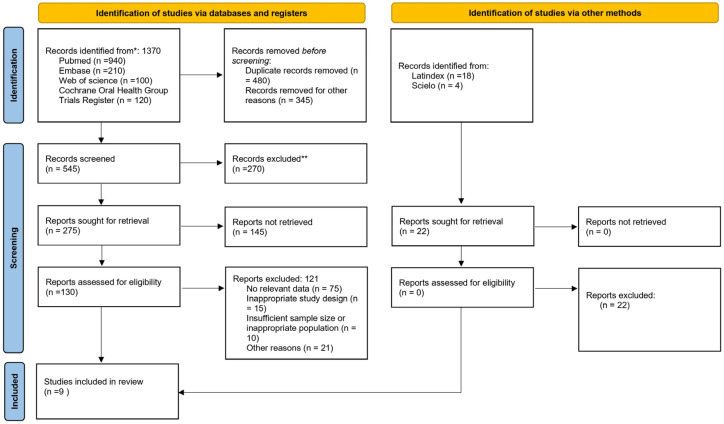
Flowchart of selected studies.

The PROSPERO database was searched in May 2025, and the protocol for this systematic review and meta-analysis was prospectively registered (registration number: CRD420251273975). This study was conducted and reported in accordance with the Preferred Reporting Items for Systematic Reviews and Meta-Analyses (PRISMA) guidelines (Figure 1) [21], along with a PRISMA 2020 Checklist (File S1).

PICO question.

Conceptual framework.

Given the conceptual distinction between clinically defined periodontitis and the detection of specific periodontal pathogens, this systematic review was structured into two analytically independent components addressing epidemiological and prognostic evidence, respectively.

Epidemiological association (primary component—meta-analysis).

Among adults aged ≥18 years (P), we investigated whether clinically defined periodontitis (I) is associated with an increased risk, incidence, or mortality of oral and/or oropharyngeal cancer compared with individuals without periodontitis (C). Eligible participants were derived from observational studies, including prospective or retrospective cohorts, case–control studies, and population-based studies. Periodontitis (gingivitis and/or periodontitis) was defined based on clinical and/or radiographic assessment or validated self-reported measures. The comparator group comprised adults without periodontitis or with a periodontally healthy status. Outcomes (O) included the risk, incidence, or mortality of oral and/or oropharyngeal cancer, reported as hazard ratios (HR), odds ratios (OR), or risk ratios (RR), with corresponding 95% confidence intervals.

Prognostic component (secondary).

In adult patients diagnosed with oral and/or oropharyngeal cancer (P), we explored whether the presence or higher burden of tumor-associated oral periodontal pathogens (I) is associated with poorer clinical outcomes compared with patients without such pathogens or with low bacterial load (C). The population included adults with histopathologically confirmed oral and/or oropharyngeal cancer. Exposure consisted of the detection and/or quantification of periodontal pathogens, including *Porphyromonas gingivalis*, *Fusobacterium nucleatum*, *Tannerella forsythia*, *Treponema denticola*, and other relevant periodontal pathogens, identified using microbiological or molecular techniques in oral/oropharyngeal tumor tissue (priority), saliva, subgingival plaque, or other biological fluids. Detection methods included PCR/qPCR, 16S rRNA gene sequencing or metagenomics, immunohistochemistry, and other validated techniques. The primary outcome (O) was overall survival (OS), while secondary outcomes included disease-free survival (DFS), progression-free survival (PFS), and recurrence-free survival (RFS).

Search strategy and database screening.

The Rayyan QCRI platform (Qatar Computing Research Institute, Doha, Qatar) was used for the identification, organization, and screening of eligible studies. A comprehensive literature search was performed across multiple electronic databases, including MEDLINE via PubMed, EMBASE via OVID, Web of Science, Scopus, Cochrane Library, ClinicalTrials.gov, and the five World Health Organization (WHO) regional bibliographic databases (AIM, LILACS, IMEMR, IMSEAR, and WPRIM). In addition, conference abstracts were explored through the Conference Proceedings Citation Index to identify potentially relevant unpublished or ongoing studies.

The search strategy was specifically tailored for each database using a combination of Medical Subject Headings (MeSH) and free-text terms related to periodontitis, oral and/or oropharyngeal cancer, and periodontal pathogens. The primary search terms included “periodontitis”, “periodontitis”, “gingivitis”, “alveolar bone loss”, “periodontal attachment loss”, “oral and/or oropharyngeal cancer”, “oropharyngeal cancer”, “head and neck squamous cell carcinoma”, “oral squamous cell carcinoma”, “*Porphyromonas gingivalis*”, “*Fusobacterium nucleatum*”, “periodontal pathogens”, and “oral microbiota”. Boolean operators (AND/OR) were applied to combine terms appropriately and optimize search sensitivity.

The electronic search was complemented by a manual review of reference lists from relevant peer-reviewed articles and reviews to identify additional eligible studies not captured through the database search. All retrieved records were imported into Rayyan QCRI, where duplicate records were removed prior to the title and abstract screening process.

Eligibility criteria.

Epidemiological association.

Inclusion criteria:

(1) Study design: Observational studies conducted in humans, including prospective or retrospective cohort studies, case–control studies, and population-based studies, published as original peer-reviewed articles.

(2) Population: Adults aged ≥ 18 years, without a previous diagnosis of oral or oropharyngeal cancer at baseline for cohort studies.

(3) Exposure: Periodontitis (gingivitis and/or periodontitis), defined by clinical and/or radiographic assessment or by validated self-reported measures; classifications based on severity, extent, or duration of periodontitis were accepted.

(4) Comparator: Individuals without periodontitis or with a periodontally healthy status.

(5) Outcomes: Risk, incidence, or mortality of oral and/or oropharyngeal cancer, reported as hazard ratios (HR), odds ratios (OR), or risk ratios (RR) with corresponding 95% confidence intervals, or with sufficient data to allow their calculation.

(6) Confounding adjustment: Studies reporting adjusted effect estimates or, at a minimum, accounting for major confounding factors such as tobacco use and/or alcohol consumption.

(7) Language and availability: Studies published in English with full-text availability.

Exclusion criteria:

(1) Study design: Cross-sectional studies without risk estimators; case series, case reports, letters to the editor, narrative or systematic reviews, editorials, commentaries, and conference abstracts.

(2) Population: Animal studies, in vitro studies, and studies conducted in pediatric or adolescent populations (<18 years).

(3) Exposure: Studies without a clear definition of periodontitis or studies evaluating oral hygiene indicators only, without a periodontal diagnosis.

(4) Outcomes: Studies without a confirmed diagnosis of oral or oropharyngeal cancer or studies reporting tumor prevalence only without a comparator group.

(5) Data availability: Studies lacking effect estimates or sufficient data to calculate them.

Prognostic component.

Inclusion criteria:

(1) Study design: Human studies, including prospective or retrospective cohort studies and translational studies with clinical follow-up; case–control studies were included only if survival outcomes were reported.

(2) Population: Adults aged ≥18 years with histopathologically confirmed oral and/or oropharyngeal cancer.

(3) Exposure: Detection and/or quantification of oral periodontal pathogens, including *Porphyromonas gingivalis*, *Fusobacterium nucleatum*, *Tannerella forsythia*, *Treponema denticola*, and other relevant periodontal pathogens, identified in tumor tissue (priority), saliva, subgingival plaque, or other biological fluids using molecular or microbiological techniques such as PCR/qPCR, 16S rRNA gene sequencing, metagenomics, immunohistochemistry, or other validated methods.

(4) Comparator: Patients with oral and/or oropharyngeal cancer without detection of periodontal pathogens or with low bacterial load.

(5) Outcomes: At least one survival outcome, including overall survival (OS), disease-free survival (DFS), progression-free survival (PFS), or recurrence-free survival (RFS), reported as hazard ratios (HR) with 95% confidence intervals or with Kaplan–Meier curves providing sufficient information for HR estimation.

(6) Language and availability: Studies published in English with full-text availability.

Exclusion criteria:

(1) Study design: Animal, in vitro, or ex vivo studies; case reports or case series without survival analyses; reviews, editorials, letters, and commentaries.

(2) Exposure: Studies assessing global oral microbiota without specific evaluation of periodontal pathogens, or studies lacking a clear description of bacterial detection methods.

(3) Outcomes: Studies without prognostic data (e.g., reporting prevalence or abundance only) or studies not allowing extraction or estimation of HRs.

(4) Methodological quality: Studies without a comparator group or with high risk of bias in key prognostic domains, as assessed by the QUIPS tool, when not justifiable.

Studies screening and data extraction.

An ad hoc standardized data extraction sheet was developed and completed independently by three investigators (LC, AOB, and BVR) using a customized data collection form. Any disagreements or uncertainties among the three investigators were resolved by three additional investigators (CR, GZ, and MR), who were unaware of the study hypothesis.

For the epidemiological association component (Arm 1), the following variables were extracted from each eligible study: first author, year of publication, country, study design, study population characteristics, periodontitis definition and assessment method, cancer site (oral cavity and/or oropharynx), effect estimate type (HR, OR, or RR), corresponding 95% confidence intervals, variables included in multivariable adjustment (with particular emphasis on tobacco and alcohol use), duration of follow-up when applicable, and main study conclusions. Effect estimates were preferentially extracted from the most fully adjusted models reported in each study. When different effect measures were reported, estimates were harmonized to allow quantitative synthesis. Extracted data were summarized in tabular form (Table 1).

For the prognostic component (Arm 2), the following variables were extracted from each eligible study: first author, year of publication, country, study design, study population, cancer type, method of pathogen detection, type of biological sample, periodontal pathogen evaluated, definition of the comparator group, survival outcome assessed, statistical analysis method, multivariable adjustment, and main study conclusions. Survival outcomes were preferentially extracted as hazard ratios (HRs) with corresponding 95% confidence intervals, derived from multivariable Cox proportional hazards models when available. Extracted data were summarized in tabular form (Table 2).

Assessment of risk of bias (RoB).

For the epidemiological association component (Arm 1), risk of bias was independently assessed by two authors (LC and KS) using all checklist items of the Newcastle–Ottawa Scale (NOS) for observational studies. Any discrepancies were resolved by discussion, with arbitration by a third reviewer (MPS) when necessary. Risk of bias was evaluated across the three NOS domains: selection, comparability, and outcome. As shown in the risk-of-bias summary, most studies were judged to have low risk of bias in the selection domain, while the comparability domain presented some concerns in several studies, mainly related to incomplete adjustment for confounding factors. In the outcome domain, all studies were rated as having some concerns, and no study was classified as high risk of bias [28].

For the prognostic component (Arm 2), risk of bias was independently assessed by two authors (LC and BVR) using the Quality In Prognosis Studies (QUIPS) tool, with disagreements resolved by consensus and consultation with a third reviewer (AOB). The QUIPS assessment covered six domains: study participation, study attrition, prognostic factor measurement, outcome measurement, study confounding, and statistical analysis and reporting. Overall, the included studies showed a low to moderate risk of bias, with some concerns mainly observed in the domains related to confounding and study attrition, while no critical risk of bias was identified across studies [29].

Statistical analysis.

Qualitative analysis.

A qualitative synthesis of all included studies was conducted, focusing on the main characteristics predefined in the eligibility criteria and extracted during the data collection process. Two analytical components were established according to the study objectives: (1) the epidemiological association component (Arm 1), evaluating the relationship between periodontitis and the risk or incidence of oral and/or oropharyngeal cancer; and (2) the prognostic component (Arm 2), assessing the association between oral periodontal pathogens and clinical outcomes in patients with oral and/or oropharyngeal cancer.

For Arm 1, qualitative analysis explored differences across studies in terms of study design, population characteristics, definitions and assessment methods of periodontitis (clinical examination, radiographic evaluation, ICD-coded diagnoses, or validated self-reported measures), cancer outcomes (oral cavity, oropharynx, tongue cancer, or OSCC), and adjustment for major confounding factors such as tobacco and alcohol consumption. Particular attention was given to the consistency of the direction of the association across studies and to methodological sources of heterogeneity, including exposure definition and population setting.

For Arm 2, qualitative synthesis focused on study design, patient characteristics, type of oral and/or oropharyngeal cancer, periodontal pathogen evaluated, biological sample analyzed, pathogen detection methods, comparator definitions, and survival outcomes assessed. The prognostic relevance of intratumoral periodontal pathogens was qualitatively evaluated based on reported associations with overall survival and other time-to-event outcomes, as well as on the degree of multivariable adjustment applied in each study.

This qualitative analysis provided the contextual framework for the subsequent quantitative synthesis and facilitated interpretation of heterogeneity across studies.

Meta-analysis.

To perform the quantitative synthesis, the included studies were grouped according to their analytical component and outcome measures. For the epidemiological association component (Arm 1), data were extracted on study identifiers, effect estimates, and corresponding measures of uncertainty. Adjusted hazard ratios (HRs), odds ratios (ORs), or risk ratios (RRs with 95% confidence intervals were preferentially extracted from the most fully adjusted models reported in each study. When different effect measures were reported, estimates were log-transformed and harmonized to allow pooling under a common metric.

For the prognostic component (Arm 2), adjusted hazard ratios (HRs) for overall survival and other time-to-event outcomes were extracted directly or estimated from Kaplan–Meier curves when necessary. All effect estimates were transformed to the logarithmic scale prior to analysis.

Meta-analyses were conducted using a random-effects model, accounting for between-study variability. The restricted maximum likelihood (REML) estimator was applied to estimate the between-study variance (τ^2^). Pooled effect estimates were calculated with 95% confidence intervals, and results were considered statistically significant at a two-sided *p*-value < 0.05. Forest plots were generated to display individual study estimates, standard errors, confidence interval limits, study weights, and the overall pooled effect, including reference lines for both the null effect and the combined effect estimate.

Statistical heterogeneity was assessed using Cochran’s Q test and quantified with the I^2^ statistic. Heterogeneity was classified as low (I^2^ < 25%), moderate (I^2^ = 25–50%), or high (I^2^ > 50%). Prediction intervals were calculated to estimate the expected range of effects in future studies.

Potential small-study effects and publication bias were evaluated through visual inspection of funnel plots, plotting effect size against standard error. Sensitivity analyses were conducted using a leave-one-out approach to assess the robustness of pooled estimates. Influence diagnostics, including Cook’s distance, DFFITS, leverage (hat values), and studentized residuals, were examined to identify influential studies or outliers.

Where applicable, trial sequential analysis (TSA) was performed using O’Brien–Fleming monitoring boundaries to assess the risk of random errors and to determine whether the accumulated evidence was sufficient in relation to the required information size.

All statistical analyses were performed using appropriate meta-analytical software, and figures were generated to support graphical interpretation of the results.

## 3. Results

The results are presented according to the two analytically independent components predefined in the study protocol. The first component (Arm 1) addresses the epidemiological association between clinically defined periodontitis and the risk of oral and/or oropharyngeal cancer. The second component (Arm 2) explores prognostic evidence regarding the association between tumor-associated periodontal pathogens and survival outcomes in patients with established oral and/or oropharyngeal cancer. These components are reported separately to avoid conceptual overlap between disease exposure and prognostic microbial factors.

Quality assessment.

**Figure 2:**
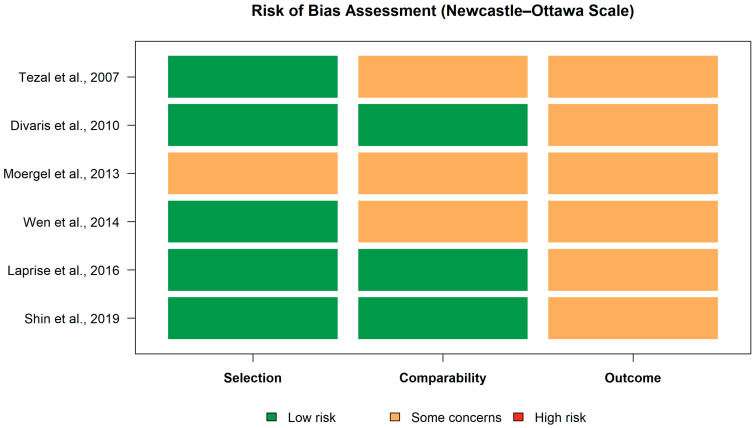
Risk of bias of included studies assessed using the Newcastle–Ottawa Scale (NOS) [10,12,15,22,23,24].

Risk of bias assessment using the Newcastle–Ottawa Scale (NOS) indicated that the majority of included studies were judged to have a low risk of bias in the selection domain. The comparability domain raised some concerns in several studies, mainly due to incomplete adjustment for relevant confounding factors, particularly smoking and alcohol consumption. In the outcome domain, all studies were rated as having some concerns, with no study classified as being at high risk of bias. Overall, the main source of bias in the epidemiological studies was related to residual confounding, whereas selection and outcome assessment domains were generally robust. The overall methodological profile of the included studies was consistent with a moderate methodological quality, which was considered acceptable for the quantitative synthesis performed (Figure 2).

Risk of bias assessment (QUIPS).

**Figure 3:**
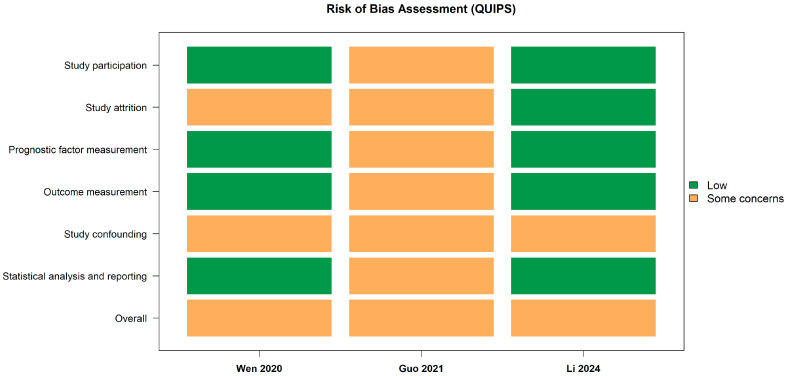
Risk of bias assessment of prognostic studies using the QUIPS tool [25,26,27].

Risk of bias assessment using the Quality In Prognosis Studies (QUIPS) tool indicated an overall low to moderate risk of bias among the included studies. Wen et al. (2020) [25] and Li et al. (2024) [27] were judged to have a low risk of bias across most domains, whereas Guo et al. (2021) [26] showed some concerns, mainly related to incomplete control of confounding factors. Across prognostic studies, the primary sources of bias were potential residual confounding and study attrition, while prognostic factor measurement, outcome assessment, and statistical analysis were generally judged at low risk. Overall, the global risk of bias was classified as “some concerns”, with no critical biases identified that would compromise the validity of the meta-analysis (Figure 3).

Association between periodontitis and risk of oral and/or oropharyngeal cancer: random-effects meta-analysis.

**Figure 4:**
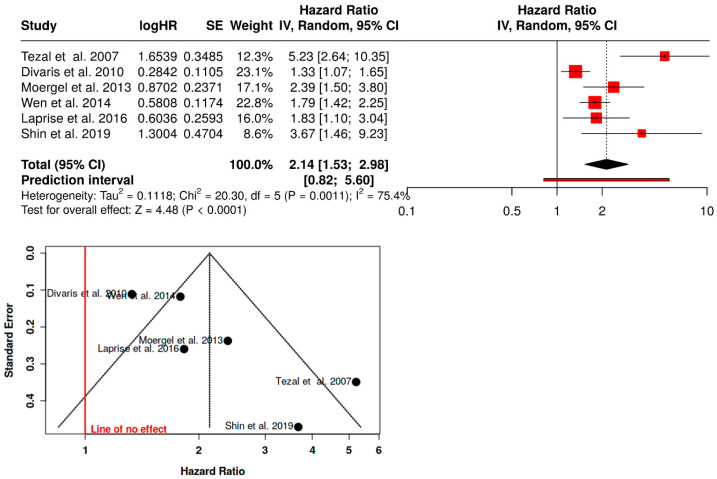
Forest plot of the random-effects meta-analysis and corresponding funnel plot assessing the association between periodontitis and the risk of oral and/or oropharyngeal cancer [10,12,15,22,23,24].

Six observational studies were included in the random-effects meta-analysis assessing the association between periodontitis and the risk of oral and/or oropharyngeal cancer. Overall, periodontitis was associated with a significantly increased risk of oral/oropharyngeal cancer (pooled HR = 2.14, 95% CI 1.53–2.98, *p* < 0.0001). Substantial between-study heterogeneity was observed (τ^2^ = 0.1118; Cochran’s Q = 20.30, df = 5, *p* = 0.0011; I^2^ = 75.4%). The prediction interval was wide (HR 0.82–5.60), indicating variability in the magnitude of the association across populations and study settings. Individual study estimates consistently favored an increased risk, with hazard ratios ranging from 1.33 to 5.23. Visual inspection of the funnel plot did not reveal marked asymmetry, although the limited number of studies and dispersion among smaller studies preclude definitive conclusions regarding publication bias (Figure 4).

Trial Sequential Analysis (TSA).

**Figure 5:**
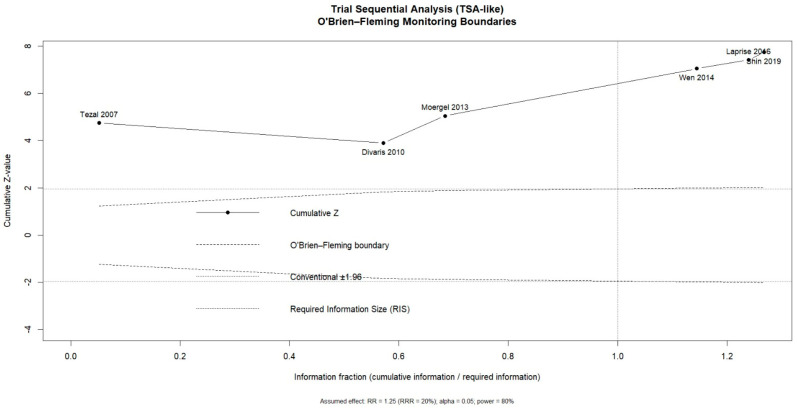
Trial sequential analysis (TSA) with O’Brien–Fleming monitoring boundaries for the association between periodontitis and oral and/or oropharyngeal cancer risk [10,12,15,22,23,24].

Trial sequential analysis (TSA) using O’Brien–Fleming monitoring boundaries showed that the cumulative Z-curve crossed both the conventional significance threshold (Z = 1.96) and the trial sequential monitoring boundaries before reaching the required information size (RIS). The cumulative inclusion of studies from 2007 to 2019 demonstrated a progressive increase in the Z-value, which remained above the monitoring boundaries in subsequent analyses despite the RIS not being fully achieved. These findings indicate that the available evidence is sufficient to support a statistically significant association between periodontitis and the risk of oral and/or oropharyngeal cancer, with a reduced risk of type I error (Figure 5).

Sensitivity analysis (leave-one-out).

**Figure 6:**
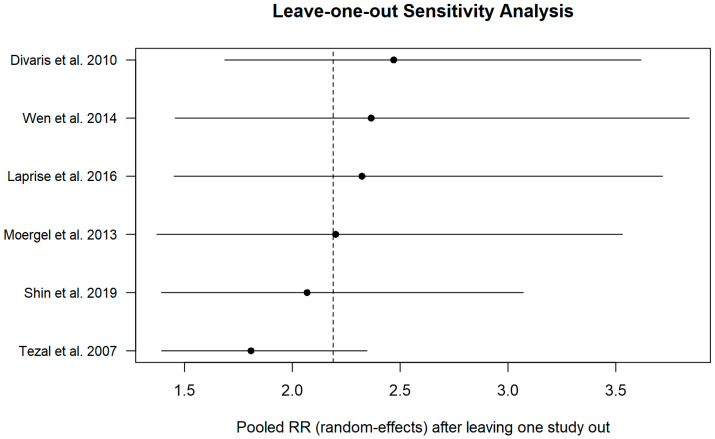
Leave-one-out sensitivity analysis of the random-effects meta-analysis assessing the association between periodontitis and oral and/or oropharyngeal cancer risk [10,12,15,22,23,24].

Sensitivity analysis using the leave-one-out approach showed that the sequential exclusion of individual studies did not substantially alter the overall effect estimate. Under the random-effects model, pooled risk estimates ranged approximately from RR ≈ 1.9 to 2.6, remaining consistently above unity across all scenarios. The removal of individual studies, including those with greater statistical weight or more extreme effect estimates, did not affect the statistical significance of the pooled result, indicating that the observed association between periodontitis and the risk of oral and/or oropharyngeal cancer is robust and not driven by any single study (Figure 6).

Influence diagnostics and outlier assessment.

**Figure 7:**
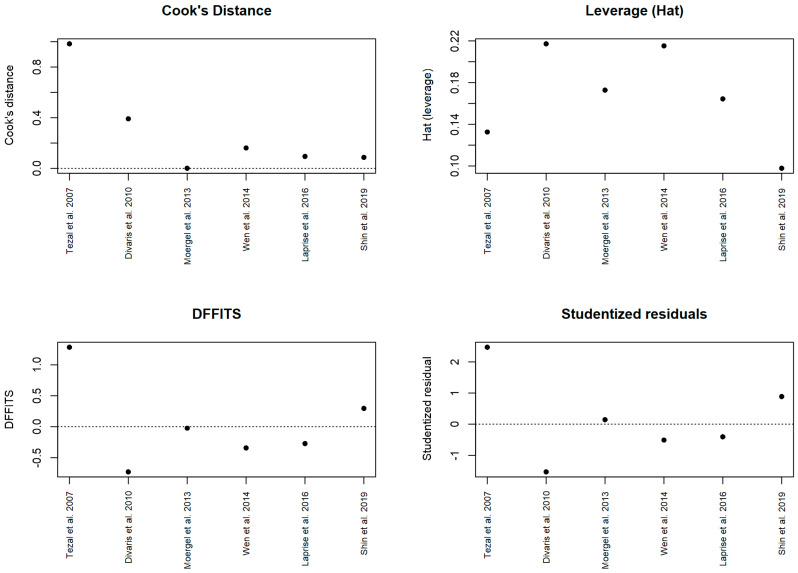
Influence diagnostics for the random-effects meta-analysis, including Cook’s distance, leverage (hat values), DFFITS, and studentized residuals [10,12,15,22,23,24].

Influence diagnostics did not identify any study exerting excessive influence on the pooled effect estimate. Cook’s distance values remained below conventional thresholds for all included studies, with no dominant observations detected. Leverage (hat) values were moderate across studies, without evidence of extreme leverage points. Consistently, DFFITS statistics did not exceed commonly accepted cutoffs, and studentized residuals were within acceptable ranges (approximately between −2 and +2), indicating the absence of influential outliers. Collectively, these findings support the stability of the pooled effect and confirm that the observed association is not driven by aberrant individual studies (Figure 7).

Meta-analysis of overall survival (OS) associated with *Porphyromonas gingivalis.*

**Figure 8:**
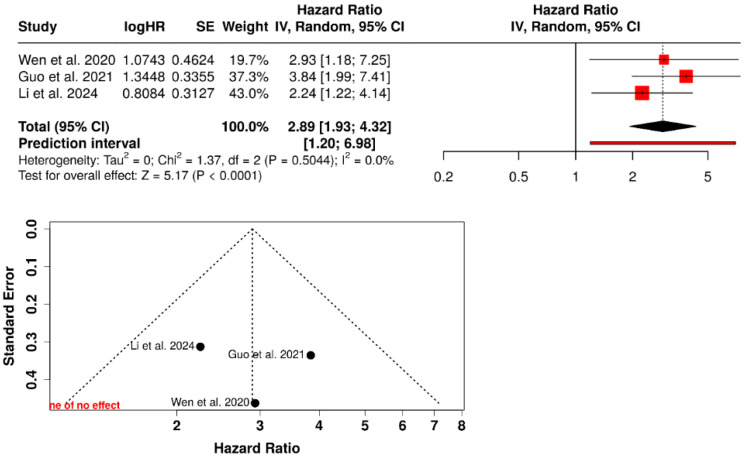
Forest plot of the random-effects meta-analysis and corresponding funnel plot evaluating the association between intratumoral *Porphyromonas gingivalis* and overall survival in oral squamous cell carcinoma [25,26,27].

Three observational studies assessed the association between intratumoral *Porphyromonas gingivalis* and overall survival (OS) in oral squamous cell carcinoma. In a random-effects meta-analysis, the presence or high expression of *P. gingivalis* was associated with an increased risk of mortality (HR = 2.89; 95% CI: 1.93–4.32; *p* < 0.0001). No heterogeneity was observed (I^2^ = 0.0%; *p* = 0.50), and the prediction interval indicated persistence of the adverse survival effect in future studies (HR: 1.20–6.98). Funnel plot inspection did not suggest relevant asymmetry, although interpretation is limited by the small number of studies (*n* = 3) (Figure 8).

Sensitivity analysis (leave-one-out).

**Figure 9:**
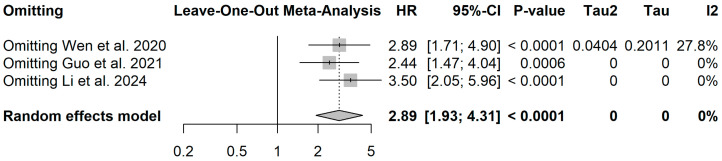
Leave-one-out sensitivity analysis of the random-effects meta-analysis evaluating the association between intratumoral *Porphyromonas gingivalis* and overall survival in oral squamous cell carcinoma [25,26,27].

Leave-one-out sensitivity analysis demonstrated that the association between intratumoral *Porphyromonas gingivalis* and overall survival remained consistent after sequential exclusion of each study. The pooled hazard ratios ranged from HR = 2.44 (95% CI: 1.47–4.04) to HR = 3.50 (95% CI: 2.05–5.96), remaining statistically significant across all scenarios. The overall effect estimate (HR = 2.89; 95% CI: 1.93–4.31) was not driven by any single study, with null to low heterogeneity observed throughout the analyses (I^2^ ≤ 27.8%), supporting the robustness of the meta-analytic findings (Figure 9).

## 4. Discussion

This discussion follows the dual-arm conceptual framework established a priori, addressing epidemiological and prognostic evidence as distinct analytical constructs. First, the epidemiological findings (Arm 1) are discussed in relation to periodontitis as a clinically defined exposure associated with oral and/or oropharyngeal cancer risk. Subsequently, prognostic findings (Arm 2) are interpreted separately, focusing on tumor-associated periodontal pathogens as microbiological factors influencing survival outcomes. This structured approach is intended to prevent conflation of etiological and prognostic mechanisms.

The results of this systematic review and meta-analysis indicate that periodontitis is consistently associated with an increased risk of oral and oropharyngeal cancer, as well as with unfavorable prognostic outcomes in patients with oral squamous cell carcinoma (OSCC). In the overall analysis, periodontitis was associated with a significant increase in the risk of oral and/or oropharyngeal cancer, with a pooled effect suggesting approximately a twofold increase in risk (HR = 2.14; 95% CI: 1.53–2.98), although with variability among the included studies. This heterogeneity likely reflects multiple methodological and clinical sources. First, periodontitis was defined using heterogeneous criteria across studies, including radiographic alveolar bone loss, full-mouth clinical periodontal examination, ICD-coded diagnoses, and validated self-reported measures, which may capture different stages and severities of disease. Second, the included studies were conducted in diverse populations with varying baseline risks, behavioral profiles, and healthcare systems, potentially influencing both periodontal status and cancer susceptibility. Finally, differences in cancer site classification (oral cavity, tongue, or oropharynx), outcome ascertainment, and diagnostic approaches may have further contributed to variability in effect estimates. Despite this heterogeneity, the direction of the association was consistent across studies, supporting the robustness of the observed relationship.

The initial evidence is derived primarily from case–control studies. In this context, Tezal et al. described a particularly strong association between radiographically assessed alveolar bone loss and the risk of tongue cancer, reporting more than a fivefold increase in risk among individuals with advanced periodontal destruction compared with those without significant bone loss (OR = 5.23; 95% CI: 2.64–10.35), even after adjustment for smoking and number of teeth [10]. Subsequently, the Carolina Head and Neck Cancer Study showed that indicators of periodontal deterioration, such as self-reported periodontitis and tooth mobility, were significantly associated with an increased risk of oral and oropharyngeal squamous cell carcinoma, even after adjustment for tobacco and alcohol consumption [30]. Consistently, Moergel et al. observed that severe chronic periodontitis was associated with an increased risk of OSCC in a European population, reinforcing the consistency of this association across different clinical settings [22].

Cohort studies provided stronger temporal support for this association. In a national cohort from Taiwan, Wen et al. demonstrated that individuals with periodontitis had a markedly higher incidence of oral and/or oropharyngeal cancer compared with those diagnosed only with gingivitis [23]. Similar findings were reported by Laprise et al. in a population from southern India, where generalized gingival recession was associated with an increased risk of oral and/or oropharyngeal cancer after adjustment for relevant behavioral and cultural factors, including tobacco, alcohol, and betel quid consumption [15]. Likewise, Shin et al. documented a notable increase in oral and/or oropharyngeal cancer risk among patients with periodontitis in a population-based cohort from South Korea, suggesting that the magnitude of the association may vary according to population characteristics and periodontal diagnostic methods [24].

Beyond epidemiological associations, recent studies indicate that specific periodontal pathogens may play a role in tumor progression. Wen et al. demonstrated that intratumoral detection of *Porphyromonas gingivalis* was associated with a significant reduction in overall survival in patients with OSCC [25]. Similarly, Guo et al. reported that high intratumoral expression of this pathogen was associated with poorer prognosis and reduced long-term survival, remaining statistically significant after multivariable adjustment [26]. More recently, Li et al. confirmed that a high intratumoral burden of *P. gingivalis* constituted an independent predictor of mortality after prolonged follow-up, even after controlling for relevant clinical and pathological factors [27]. In line with these findings, the prognostic meta-analysis performed in the present study showed that the presence or high intratumoral expression of this pathogen was associated with a significantly increased risk of mortality, with no relevant heterogeneity among the included studies.

From a methodological perspective, these results should be interpreted in light of the inherent limitations of observational studies. It has been noted that widely used quality assessment tools, such as the Newcastle–Ottawa Scale, present methodological weaknesses that may influence overall risk-of-bias estimation [28]. Likewise, methodological frameworks for assessing bias in prognostic factor studies highlight potential issues related to exposure measurement, incomplete control of confounding variables, and losses to follow-up [29]. In addition, cancer-related treatments, particularly radiotherapy and chemotherapy, may adversely affect periodontal tissues and oral health, potentially influencing periodontal status and complicating the interpretation of associations observed in observational and prognostic studies.

Recent clinical evidence further supports this perspective. A cross-sectional study by Abou-Bakr et al. (2025) [30] reported a high frequency of periodontitis among head and neck cancer patients after radiotherapy, highlighting the substantial periodontal burden in treated patients and reinforcing the relevance of periodontal assessment during survivorship care.

The heterogeneity observed among the included studies may be explained, at least in part, by differences in the clinical definition of periodontitis, the severity of exposure, and the systemic or genetic characteristics of the analyzed populations, a phenomenon previously described in other areas of biomedical research [31].

From a clinical perspective, these findings suggest that periodontal status may represent a modifiable factor within oral cancer prevention and management strategies. Periodontal screening could be considered as part of a comprehensive risk assessment in populations at increased risk for oral and/or oropharyngeal cancer, while adequate periodontal care may support overall oral health in patients undergoing cancer treatment. However, current evidence does not support causal inferences, and integration into formal prevention or management protocols should be guided by future prospective and interventional studies.

From a biological perspective, there is growing evidence supporting a link between periodontitis and cancer. The microbiome has been identified as a relevant modulator of oncogenic risk due to its influence on persistent inflammation, immune response, and epithelial integrity [32]. In this context, sustained interactions between periodontal pathogens and the host immune system may promote local conditions that facilitate tumor development.

Advances in translational research in this field require academic environments that promote effective mentorship and interdisciplinary collaboration, which are essential to ensure the quality and reproducibility of scientific output [33]. From a clinical standpoint, photodynamic therapy has been proposed as a potential adjunctive approach to subgingival mechanical instrumentation (etiological therapy) in the management of periodontitis. However, its clinical usefulness remains limited, and current practical treatment guidelines do not support its routine use in periodontal therapy. Therefore, photodynamic therapy should be considered only as an adjunct to conventional mechanical treatment and not as a standard or standalone intervention, and its direct impact on oncological outcomes has not yet been clearly established [34].

At the immunological level, certain bacterial pathogens have been shown to induce apoptosis and alter dendritic cell function, thereby affecting the regulation of immune responses [35]. Additionally, systemic factors such as prolonged exposure to glucocorticoids have been associated with alterations in bone and inflammatory homeostasis [36], which may indirectly influence tissue susceptibility and the progression of chronic diseases.

Cancer-related inflammation represents a central component of tumor biology [37]. In this regard, periodontitis may be considered a persistent source of chronic inflammation that, in combination with other risk factors, contributes to the development and progression of oral and/or oropharyngeal cancer.

Finally, progress in this field will benefit from more standardized and collaborative research designs. Initiatives focused on systematic registration and collection of clinical data have proven useful in improving methodological quality and comparability of results in complex clinical research [38]. Future prospective studies integrating standardized periodontal assessment, microbiological and immunological analyses, and long-term clinical follow-up will be essential to elucidate the underlying mechanisms and to determine the true impact of periodontal prevention and therapy on oncological outcomes.

In addition, an important limitation of the existing literature relates to the lack of uniformity in periodontitis case definitions across studies. The use of standardized and currently accepted diagnostic criteria, in accordance with the contemporary periodontal classification system established by the 2017 World Workshop, is essential to ensure consistency and comparability across epidemiological and prognostic research. The application of structured frameworks, such as the ACES system, to operationalize the 2018 classification in population-based studies may substantially reduce methodological heterogeneity and improve the interpretability of future findings [39,40].

## 5. Conclusions

This systematic review and meta-analysis demonstrate a consistent epidemiological association between periodontitis and oral and/or oropharyngeal cancer risk. Exploratory prognostic evidence suggests that tumor-associated *Porphyromonas gingivalis* may be linked to adverse survival outcomes; however, these findings are based on limited observational data and should be interpreted with caution.

## Figures and Tables

**Table 1:** Characteristics of observational studies included in the epidemiological association component (Arm 1).

Author (Year)	Country	Study Design	Population	Periodontitis Definition	Cancer Site	Adjusted Effect Estimate (HR/OR/RR)	Key Confounders Adjusted	Main Result
Tezal et al. (2007) [10]	USA	Case–control	Adult men without prior cancer	Alveolar bone loss measured radiographically	Tongue cancer	OR = 5.23 (95% CI 2.64–10.35)	Age, smoking status, number of teeth	Increasing alveolar bone loss was strongly associated with higher tongue cancer risk.
Divaris et al. (2010) [12]	USA	Case–control	Adults with head and neck SCC	Self-reported periodontitis and tooth mobility	Oral/oropharyngeal cancer	OR = 1.33 (95% CI 1.07–1.65)	Age, sex, smoking, alcohol	Periodontitis indicators were associated with increased oral and/or oropharyngeal cancer risk.
Moergel et al. (2013) [22]	Germany	Case–control	Adults with OSCC and controls	Clinical periodontal examination	Oral squamous cell carcinoma	OR = 2.39 (95% CI 1.50–3.80)	Smoking, alcohol	Severe periodontitis was significantly associated with OSCC.
Wen et al. (2014) [23]	Taiwan	Nationwide cohort	Adults ≥ 20 years	ICD-coded periodontitis vs. gingivitis	Oral and/or oropharyngeal cancer	HR = 1.79 (95% CI 1.42–2.25)	Age, sex, comorbidities	Periodontitis was associated with increased oral and/or oropharyngeal cancer incidence.
Laprise et al. (2016) [15]	India	Case–control	Adults with incident oral and/or oropharyngeal cancer	Clinical gingival recession and inflammation	Oral and/or oropharyngeal cancer	OR = 1.83 (95% CI 1.10–3.04)	Tobacco, alcohol, betel quid, SES	Generalized gingival recession increased oral and/or oropharyngeal cancer risk.
Shin et al. (2019) [24]	South Korea	Population-based cohort	Adults ≥ 40 years	Periodontitis diagnosis from health records	Oral and/or oropharyngeal cancer	HR = 3.67 (95% CI 1.46–9.23)	Age, sex, smoking, alcohol	Periodontitis was associated with a markedly increased oral and/or oropharyngeal cancer risk.

**Table 2:** Characteristics of studies included in the prognostic component (Arm 2) assessing the association between intratumoral periodontal pathogens and overall survival in oral squamous cell carcinoma.

Author (Year)	Country	Study Design	Population	Cancer Type (OSCC)	Periodontal Pathogen (Exposure)	Sample Type	Pathogen Evaluated	Comparator	Outcome (OS)	Statistical Analysis	Main Conclusion
Wen et al. (2020) [25]	China	Retrospective cohort	Adults with histopathologically confirmed diagnosis	OSCC	Immunohistochemistry (IHC)	Tumor tissue	*Porphyromonas gingivalis*	Pg-positive vs. Pg-negative	Overall survival (OS)	Cox proportional hazards model	Intratumoral presence of *P. gingivalis* was associated with a significant reduction in overall survival.
Guo et al. (2021) [26]	China	Retrospective cohort	Adults with OSCC undergoing surgery	OSCC	Immunohistochemistry (IHC)	Tumor tissue	*Porphyromonas gingivalis*	High vs. low expression	Overall survival (OS)	Cox proportional hazards model	High intratumoral expression of *P. gingivalis* was associated with poorer prognosis and reduced long-term survival.
Li et al. (2024) [27]	China	Retrospective cohort	Adults with OSCC (long-term follow-up)	OSCC	PCR + Immunohistochemistry	Tumor tissue	*Porphyromonas gingivalis*	Strong vs. weak expression	Overall survival (OS)	Cox proportional hazards model	High intratumoral burden of *P. gingivalis* was an independent predictor of worse overall survival after multivariable adjustment.

## Data Availability

The raw data supporting the conclusions of this article will be made available by the authors, without undue reservation.

## Supplementary Materials

The following supporting information can be downloaded at: https://www.mdpi.com/article/10.3390/dj14020080/s1, File S1: PRISMA 2020 Checklist.
